# R-spondin-1 induces Axin degradation via the LRP6-CK1ε axis

**DOI:** 10.1186/s12964-023-01456-y

**Published:** 2024-01-05

**Authors:** Lifeng Tan, Mengfang Yan, Zijie Su, Hanbin Wang, Huan Li, Xibao Zhao, Shanshan Liu, Long Zhang, Qi Sun, Desheng Lu

**Affiliations:** 1 Guangdong Provincial Key Laboratory of Regional Immunity and Disease, International Cancer Center, Marshall Laboratory of Biomedical Engineering, Department of Pharmacology, Shenzhen University Medical School, Shenzhen University, Shenzhen, 518055 Guangdong China; 2 Department of Research, The Affiliated Tumor Hospital of Guangxi Medical University, Nanning, 530021 China; 3 MOE Laboratory of Biosystems Homeostasis & Protection and Innovation Center for Cell Signaling Network, Life Sciences Institute, Zhejiang University, Hangzhou, 310058 China

**Keywords:** Axin, CK1ε, LRP6, RSPO1, Wnt/β-catenin signaling

## Abstract

R-spondins (RSPOs) are secreted signaling molecules that potentiate the Wnt/β-catenin pathway by cooperating with Wnt ligands. RSPO1 is crucial in tissue development and tissue homeostasis. However, the molecular mechanism by which RSPOs activate Wnt/β-catenin signaling remains elusive. In this study, we found that RSPOs could mediate the degradation of Axin through the ubiquitin–proteasome pathway. The results of Co-IP showed that the recombinant RSPO1 protein promoted the interaction between Axin1 and CK1ε. Either knockout of the CK1ε gene or treatment with the CK1δ/CK1ε inhibitor SR3029 caused an increase in Axin1 protein levels and attenuated RSPO1-induced degradation of the Axin1 protein. Moreover, we observed an increase in the number of associations of LRP6 with CK1ε and Axin1 following RSPO1 stimulation. Overexpression of LRP6 further potentiated Axin1 degradation mediated by RSPO1 or CK1ε. In addition, recombinant RSPO1 and Wnt3A proteins synergistically downregulated the protein expression of Axin1 and enhanced the transcriptional activity of the SuperTOPFlash reporter. Taken together, these results uncover the novel mechanism by which RSPOs activate Wnt/β-catenin signaling through LRP6/CK1ε-mediated degradation of Axin.

**Graphical Abstract:**

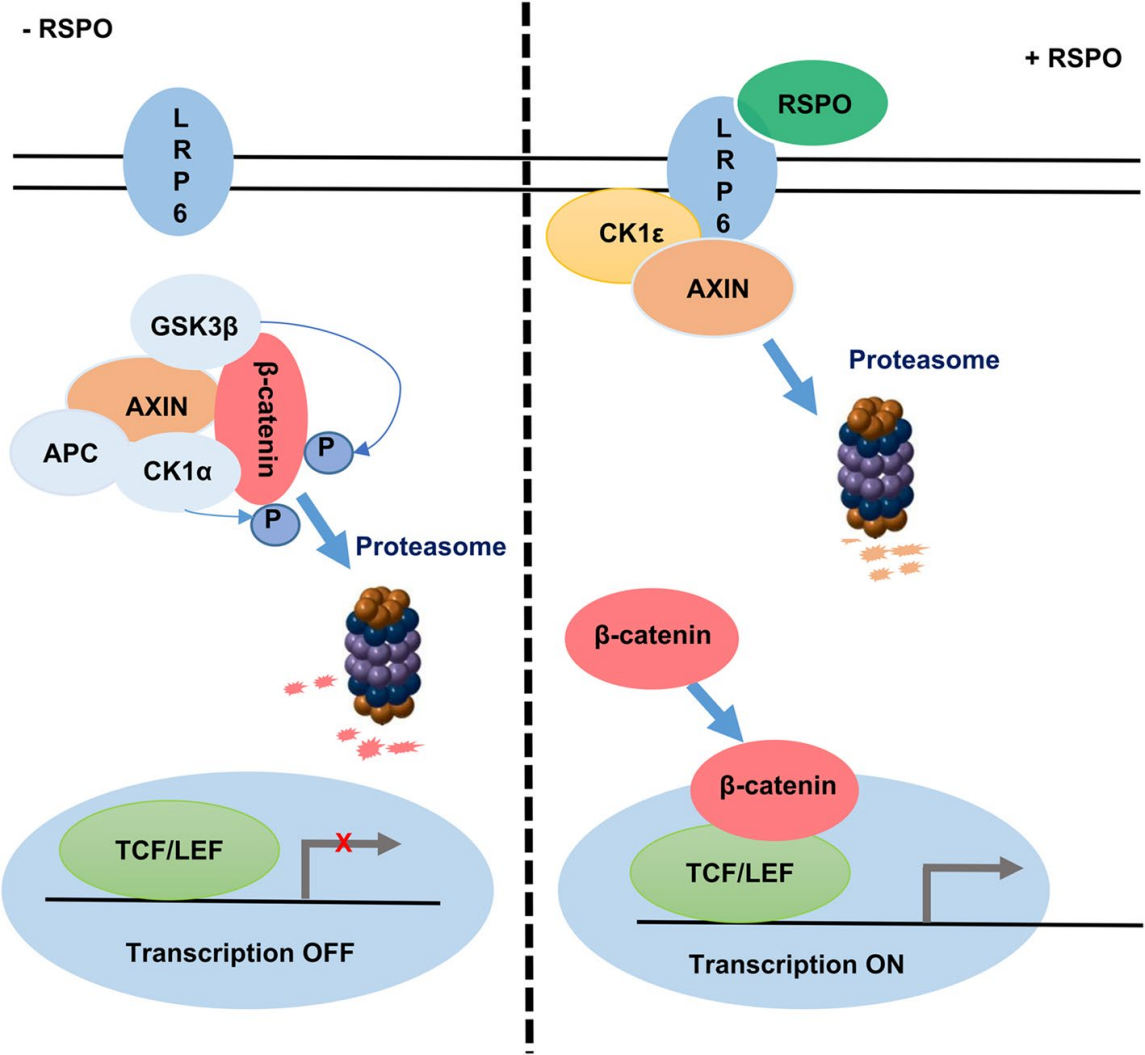

**Supplementary Information:**

The online version contains supplementary material available at 10.1186/s12964-023-01456-y.

## Introduction

R-spondin proteins (RSPOs) are secreted agonists of the Wnt signaling pathway. The RSPO protein family consists of four homologous members, including RSPO1, RSPO2, RSPO3 and RSPO4, which share 60% amino acid sequence homology [1]. All RSPO family members share the following conserved domain pattern: an N-terminal signal peptide, two adjacent furin-like cysteine-rich domains (FU1 and FU2), a thrombospondin (TSP) domain and a basic amino acid rich (BR) domain at the C-terminus [2]; of these domains, the FU1 and FU2 domains lie close to the N-terminus and are solely responsible for Wnt signaling activation [3]. RSPO molecules bind to the G-protein coupled receptor (LGR) family (LGR4/5/6) [4, 5] and the cell-surface transmembrane E3 ubiquitin ligases, zinc and ring finger 3 (ZNFR3) and its homolog ring finger protein 43 (RNF43) [6]. This binding induces membrane clearance of the ubiquitin ligases ZNRF3/RNF43 and accumulation of the Wnt receptors Frizzled and LRP5/6 on the plasma membrane of cells, thereby increasing the sensitivity of cells to Wnt ligands and increasing Wnt/β-catenin signaling [6]. RSPO proteins may also activate the Wnt signaling pathway through other mechanisms, such as antagonizing DKK1 and interacting with the LRP6 receptor [7, 8].

RSPO1 plays important roles in the homeostasis of multiple tissues, including the skin, intestine and muscle [9–11]. RSPO1 exhibits mitogenic activity in stem cells and may have potential therapeutic uses in regenerative medicine [10, 12, 13]. Chen et al. reported that recombinant RSPO1 could promote hair regeneration by targeting the Wnt/β-catenin signaling pathway [14]. RSPO1 was found to be highly expressed in ovarian cancer cells and tissues and modulated ovarian cancer biological activity by activating Wnt/β-catenin signaling [15]. Recently, a gain-of-function RSPO1 mutation (p.R219W) was identified as a genetic trigger that drives obesity in response to high-fat diets [16].

Axin is a crucial scaffolding protein that is responsible for the assembly of the destruction complex of β-catenin. Axin directly interacts with β-catenin, casein kinase 1α (CK1α), glycogen synthase kinase 3β (GSK3β) and adenomatous polyposis coli (APC) and scaffolds the destruction complex to promote β-catenin degradation [17]. Upon Wnt stimulation, Wnt receptors recruit Axin and the GSK3β complex to form a high-molecular-weight LRP signalosome [18]. Following the recruitment of Axin to the LRP signalosome, the β-catenin destruction complex is disassembled, and stabilized β-catenin is translocated to the nucleus, where it interacts with TCF/LEF transcription factors and activates the transcription of Wnt target genes [19].

Casein kinase 1ε (CK1ε) is a member of the serine/threonine CK1 family, which is involved in many cellular processes, including regulation of the Wnt signaling pathway, circadian rhythm, cell division and DNA repair [20–22]. The CK1 family contains seven members, including α, β1, γ1, γ2, γ3, δ, and ε [21]. As a critical regulator, CK1ε can phosphorylate and regulate multiple targets in the Wnt/β-catenin signaling cascade, including dishevelled (DVL) [23, 24], low-density lipoprotein receptor-related protein 6 (LRP6) [25], APC [23, 24, 26], Axin [24] and amino-terminal enhancer of split (AES) [27]. In our recent study, we showed that transmembrane protein 97 (TMEM97) promoted Wnt/β-catenin signaling by potentiating the association between LRP6 and CK1δ/ε, which is implicated in the tumorigenesis of breast cancer [28].

In this study, we found that RSPOs could destabilize Axin and induce its degradation. We further explored the underlying mechanism of RSPO-mediated degradation of Axin1. Our results revealed that RSPO induced the degradation of Axin1 through a LRP6/CK1ε-mediated mechanism.

## Materials and methods

### Reagents and antibodies

Recombinant human RSPO1 protein (rRSPO1) was purchased from Novo protein (CX83; Shanghai, China), and recombinant Wnt3A protein (rWnt3A) was purchased from R&D Systems (5036-WN-010; Minneapolis, MN, USA). SR3029 was purchased from MedChemExpress (HY-100011; MCE, Monmouth Junction, NJ, USA). MG132 (133407–82-6; Saint Louis, Missouri, USA) was purchased from Sigma–Aldrich. The following primary antibodies were used: anti-CK1δ (sc-55553), anti-Ub (sc-271289) and anti-β-catenin (sc-7963), which were purchased from Santa Cruz Biotechnology; anti-V5 (#13202), anti-LRP6 (#2560), anti-p-LRP6 (Ser1490) (#2568), anti-Axin1 (#2087) and anti-Axin2 (#2151), which were purchased from Cell Signaling Technology; anti-GFP (66002–1-Ig), anti-Flag (20543–1-AP), anti-IgG (30000–0-AP) and anti-GAPDH (600004–1-Ig), which were purchased from Proteintech; anti-CK1ε (ab270997) was purchased from Abcam; and anti-β-actin (AC026), which was purchased from ABclonal.

### Plasmids

The expression plasmids of C-terminally Flag-tagged and His-tagged RSPO1, RSPO2, and RSPO3 were purchased from Weizhen Bioscience Inc. (Beijing, China). The SuperTOPFlash reporter plasmid was a gift from the University of California, San Diego by Karl Willert. For construction of the C-terminally V5-tagged LRP6 and V5-tagged Axin1 expression plasmids, the cDNAs encoding LRP6 and Axin1 were amplified by PCR. The PCR products of LRP6 and Axin1 were subcloned and inserted into the pcDNA 3.1/V5-His mammalian expression vector. For the construction of GFP-tagged CK1ε, the cDNAs encoding CK1ε were amplified by PCR, subcloned, and inserted into the pEGFP-N1 expression vector (Weizhen Bioscience).

### Cell culture

Human embryonic kidney 293 T (HEK293T) cells (ATCC, Manassas, VA, USA) were grown in Dulbecco’s modified Eagle’s medium (DMEM) containing 10% fetal bovine serum (FBS) and 1% penicillin–streptomycin in an incubator at 37 °C with approximately 95% humidity and 5% CO_2_.

### Generation of knockout cell lines by CRISPR–Cas9

CK1ε/δ and LRP6 were knocked out in HEK293T cells by using CRISPR/Cas9 technology. The sgRNA sequences of CK1ε/δ and LRP6 (Table 1) were cloned and inserted into the LentiCRISPRv2 vector (Addgene, Cambridge, MA, USA), and the CK1ε/δ and LRP6 CRISPR/Cas9 KO plasmids were obtained. Lentivirus was produced by using 10 μg CRISPR/Cas9 KO plasmid or control CRISPR/Cas9 plasmid, 2.5 μg pMD2. G (Addgene, Cambridge, MA, USA) and 7.5 μg psPAX2 (Addgene, Cambridge, MA, USA) in HEK293T cells with 50 μL Lipofectamine 2000 transfection reagent under standard conditions. The medium was changed after 16–18 h of transfection. The first supernatant was harvested after 24 h, and then fresh medium was added to the cells. A second wave of supernatant was collected at 24-hour intervals and then centrifuged at 20,000 rpm for 2 h at 4 °C to harvest virus particles. Virus and 8 μg/mL polybrene were added to HEK293T cells to infect the cells. At 72 h after infection, the cells were selected with exposure to puromycin (A11138–03; Thermo Fisher Scientific, Waltham, MA, USA) for 7–14 days. Then, the cells were harvested, and CK1ε/δ or LRP6 deficiency was confirmed by Western blotting.

**Table 1:** Primer sequences

Gene	Primer sequences
sgCK1ε	GCAGGTGCCAACATCGCCTC
sgCK1δ	ATGACGCCGAGCGGGAGCGC
sgLRP6-F	CACCGTGCCATAGATTACGATCCTG
sgLRP6-R	AAACCAGGATCGTAATCTATGGCAC
Axin1-RT-F	GGTTTCCCCTTGGACCTCG
Axin1-RT-R	CCCGTCGAAGTCTCACCTTTAATG
C-JUN-RT-F	TCCAAGTGCCGAAAAAGGAAG
C-JUN-RT-R	CGAGTTCTGAGCTTTCAAGGT
DKK1-RT-F	GGAATAAGTACCAGACCATTGACAAC
DKK1-RT-R	GGGACTAGCGCAGTACTCATCA
AXIN2-RT-F	CAACACCAGGCGGAACGAA
AXIN2-RT-R	GCCCAATAAGGAGTGTAAGGACT
GAPDH-RT-F	CCAGAACATCATCCCTGCCTCTACT
GAPDH-RT-R	GGTTTTTCTAGACGGCAGGTCAGGT

### Luciferase reporter assays

HEK293T cells in 24-well plates were transfected with the SuperTOPFlash reporter plasmid, the control plasmid for β-gal and the indicated amounts of expression plasmids using Lipofectamine 2000 according to the manufacturer’s instructions. At 48 h after transfection, the luciferase assay was performed using a luciferase assay kit (E1501; Promega, Shanghai, China) according to the manufacturer’s protocol. The results are presented as the mean ± S.D. of at least three independent experiments. The luciferase activity was expressed as fold induction relative to the control.

### Immunoblotting and coimmunoprecipitation

The cells were lysed with RIPA lysis buffer (50 mM Tris-HCl, pH 7.4, 150 mM NaCl, 1% Nonidet P-40, 0.1% SDS, 0.5% sodium deoxycholate, and 1 mM EDTA) together with the protease inhibitor phenylmethylsulfonyl fluoride, followed by sonication. A BCA protein assay kit (P0009; Beyotime, Shanghai, China) was used to quantify the protein concentration. Equal amounts of proteins were then separated by SDS–PAGE in an 8% sodium dodecyl sulfate (SDS)-polyacrylamide gel followed by transfer to polyvinylidene difluoride membranes (PVDF, Millipore, Burlington, MA, USA). Western blotting was performed at 4 °C overnight with primary antibodies. After blocking with 5% nonfat powdered milk (Sangon Biotech) at room temperature for 1 h, the PVDF membranes were then incubated for another hour at room temperature with the relevant HRP-conjugated goat anti-mouse or anti-rabbit IgG secondary antibodies. The blots were visualized by a Tanon5200 Chemiluminescent Imaging System (Tanon, Shanghai, China) or X-ray film (Kodak, Rochester, NY) after incubation with ECL Plus Western Blotting Substrate (Thermo Fisher Scientific). The outcomes of the fluorescence reaction were then analyzed by ImageJ 1.8.0 Analysis Software (National Institutes of Health).

For coimmunoprecipitation, cells were lysed with a RIPA lysis mixture containing protease inhibitor (B14001; Selleckchem, Shanghai, China), phosphatase inhibitors (B15001; Bimake, Beijing, China), and PMSF. Lysates were collected and then centrifuged at 15,000 rpm at 4 °C for 15 min. The supernatant was incubated overnight with specific primary antibody or control IgG at 4 °C and then incubated with protein A/G magnetic beads (B23202; Bimake, Beijing, China) for 3 h. The magnetic beads were washed 3 times with RIPA lysis buffer. Proteins were eluted by boiling the sample in SDS loading buffer and analyzed by SDS–PAGE and Western blotting.

### Real-time quantitative PCR analyses

RNA was extracted with RNAiso Plus (#9109; Takara, Beijing, China) and then reverse-transcribed with a PrimeScript RT kit (RR036B; Takara, Beijing, China) according to the manufacturer’s protocol. Quantitative PCR analysis was performed using 2 × SYBR GREEN QPCR Master Mix (LS2062; Promega, Shanghai, China). The primer pairs used for quantitative PCR amplification are indicated in Table 1.

### Statistical analyses

Data are presented as the mean ± standard deviation of the mean. All experiments were independently replicated at least three times. Statistical analyses were performed using GraphPad Prism 9.5. A *P* value < 0.05 was considered to indicate statistical significance.

## Results

### RSPO family members downregulate the protein expression of Axin1

**Fig. 1:**
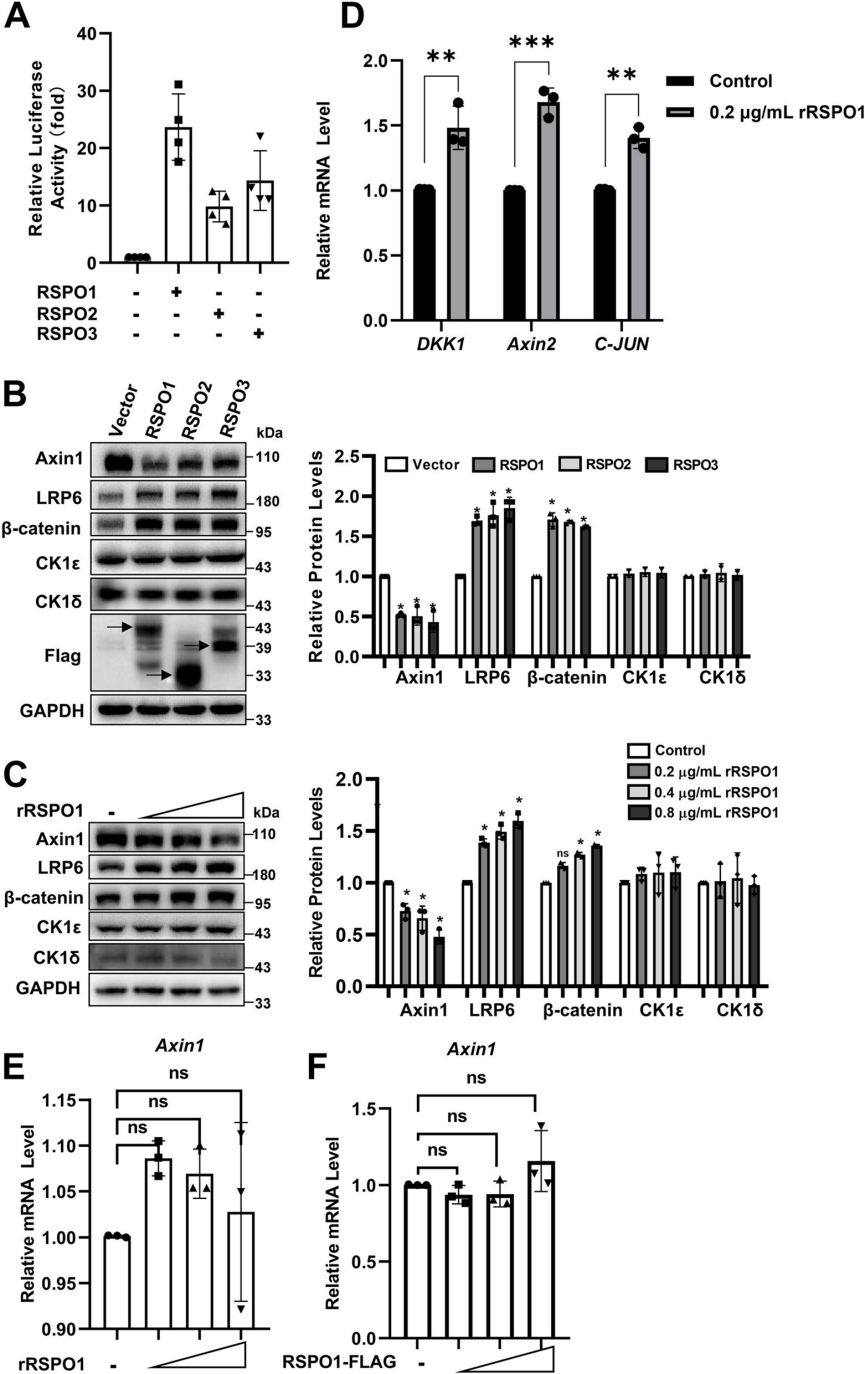
RSPOs mediate Axin1 degradation at the protein level. **a** The SuperTOPFlash luciferase reporter gene was cotransfected into HEK293T cells with expression plasmids encoding RSPO1-Flag, RSPO2-Flag and RSPO3-Flag. The luciferase values were normalized to β-gal activity and expressed as fold induction relative to the basal activity. Each treatment was performed in four replicates. **b** HEK293T cells were transfected with empty vector or RSPO1-Flag, RSPO2-Flag and RSPO3-Flag expression plasmids. The protein expression levels of endogenous Axin1, LRP6, β-catenin, CK1δ and CK1ε were determined by Western blotting. The data shown are representative of three independent experiments. The protein levels from Western blotting in Panel B were quantitated by densitometry and normalized to GAPDH. **c** HEK293T cells were treated with recombinant RSPO1 protein (rRSPO1) (0.2, 0.4 and 0.8 μg/mL) for 12 h. The protein expression levels of endogenous Axin1, LRP6, β-catenin, CK1δ and CK1ε were measured by Western blotting. The data shown are representative of three independent experiments. The protein levels from Western blotting in Panel D were quantitated by densitometry and normalized to GAPDH. **d** HEK293T cells were treated with 0.2 μg/mL rRSPO1 for 12 h before harvesting. RT–qPCR was performed to measure the mRNA expression of Wnt target genes (*DKK1*, *Axin2* and *c-JUN*). **e** HEK293T cells were treated with rRSPO1 (0.2, 0.4 and 0.8 μg/mL) for 12 h, and the mRNA level of *Axin1* was determined by RT–qPCR. **f** HEK293T cells were transfected with gradient concentrations of the indicated plasmids encoding Flag-tagged RSPO1. RT–qPCR was performed to measure *Axin1* mRNA expression. Data are presented as the mean ± SEM, *p < 0.05, **p < 0.01, ***p < 0.01, *n* = 3

RSPOs potentiate Wnt/β-catenin signaling by stabilizing the Wnt receptors Frizzled and LRP5/6 on the plasma membrane [6]. We investigated the effects of RSPOs on the Wnt/β-catenin signaling pathway. A SuperTOPFlash reporter was transfected into HEK293T cells together with RSPO1, RSPO2, and RSPO3 expression plasmids. The expression of all three RSPOs significantly enhanced the transcriptional activity of the SuperTOPFlash reporter (Fig. 1a). In addition, we examined the effect of the RSPOs on important components of the Wnt pathway. As expected, all three RSPOs increased the protein expression of LRP6 and β-catenin but had little effect on the expression of CK1δ and CK1ε (Fig. 1b). Surprisingly, overexpression of all three RSPOs dramatically reduced the level of Axin1 protein (Fig. 1b). We also compared the effect of recombinant RSPO1, RSPO2 and RSPO3 proteins on Axin1 expression in HEK293T cells. As shown in Supplementary Fig. 1, recombinant RSPO1 protein exerts a slightly stronger effect than RSPO2 and RSPO3. Thus, the recombinant RSPO1 protein was selected for further study.

To validate the effect of the RSPOs on Axin1 expression, HEK293T cells were treated with different doses of recombinant RSPO1 protein. As demonstrated in Fig. 1c, treatment with recombinant RSPO1 dose-dependently decreased Axin1 protein expression and increased the protein levels of LRP6 and β-catenin, while recombinant RSPO1 did not affect the expression of CK1δ and CK1ε (Fig. 1c). Consistently, real-time PCR analysis showed that recombinant RSPO1 increased the mRNA expression of *DKK1*, *Axin2*, and *c-JUN* in HEK293T cells (Fig. 1d).

We examined the effect of recombinant RSPO1 and the overexpression of RSPO1 on the mRNA expression of *Axin1* in HEK293 cells. Neither recombinant RSPO1 nor overexpression of RSPO1 had any significant influence on the mRNA expression of *Axin1* (Fig. 1e-f), indicating that RSPO1-induced downregulation of Axin1 expression was independent of transcriptional regulation.

### RSPO1 mediates Axin1 degradation through the ubiquitin–proteasome pathway

**Fig. 2:**
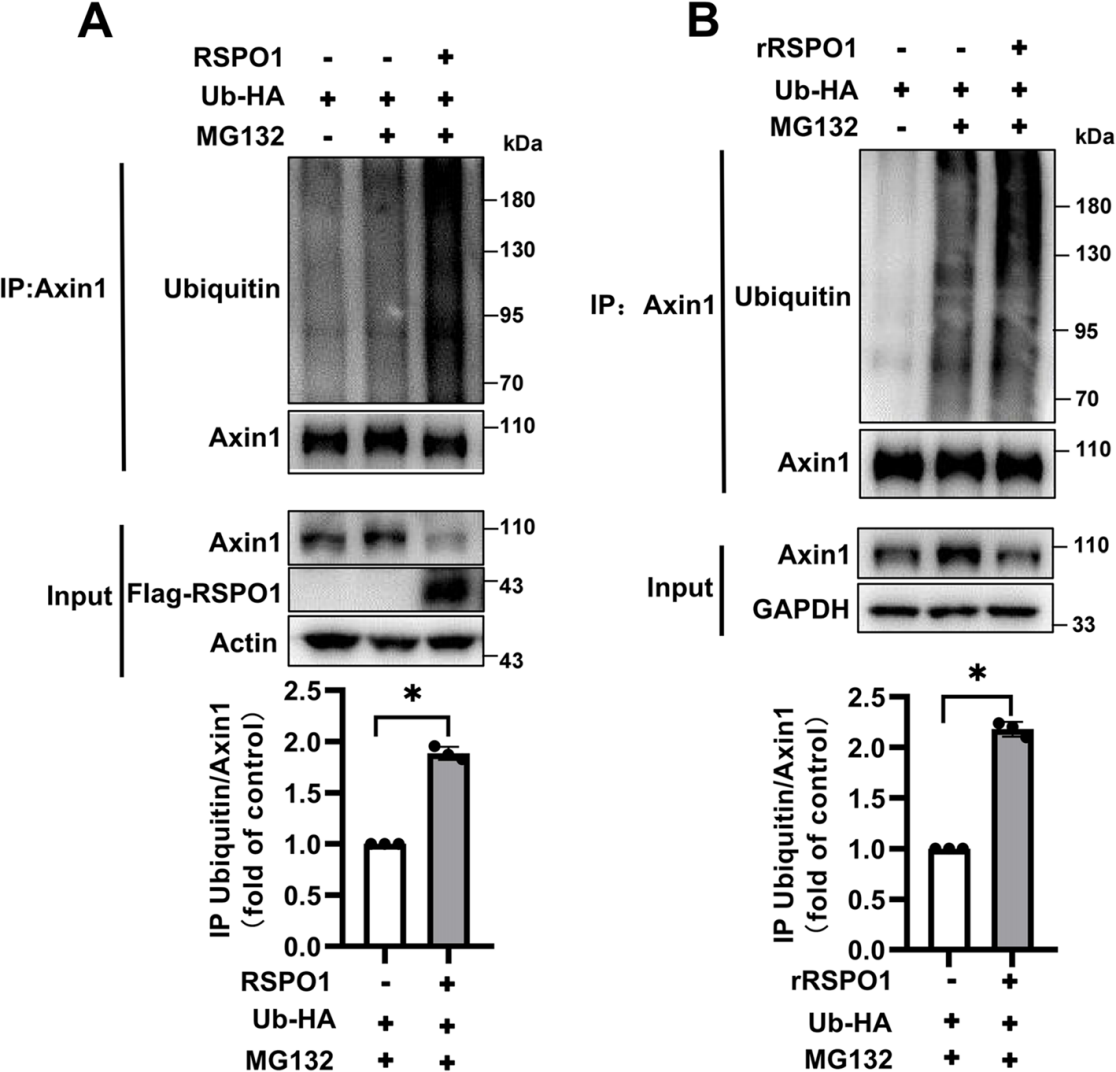
RSPO1 mediates Axin1 degradation through the ubiquitin–proteasome pathway. **a** The expression plasmids for Ub-HA and RSPO1-Flag were transfected into HEK293T cells, the cells were treated with 10 μM MG132 for 8 h before collection, and the ubiquitination levels of endogenous Axin1 were determined by Western blotting. The data shown are representative of three independent experiments. The levels of polyubiquitinated Axin1 were quantitated by densitometry and normalized to Axin1. Quantitative results are displayed at the bottom. **b** HEK293T cells were treated with 0.2 μg/mL rRSPO1 alone or in combination with 10 μM MG132 for 8 h, and the ubiquitination level and protein expression level of endogenous Axin1 were determined by immunoblotting. The data shown are representative of three independent experiments. The levels of polyubiquitinated Axin1 were quantitated by densitometry and normalized to Axin1. Quantitative results are displayed at the bottom. Data are presented as the mean ± SEM, *p < 0.05, **p < 0.01, ***p < 0.01, *n* = 3

As the ubiquitin–proteasome system is the major intracellular degradation system for protein turnover in the cell, we determined whether RSPO1-mediated Axin1 degradation was associated with the ubiquitin–proteasome pathway. HEK293T cells were transfected with an expression vector encoding HA-Ubiquitin. Treatment with the proteasome inhibitor MG132 increased Axin1 expression. Polyubiquitinated Axin1 was detected in high-molecular-weight smear bands in the presence of MG132. Either recombinant RSPO1 or expression of RSPO1-Flag decreased the levels of Axin1 protein and further increased the polyubiquitination of Axin1 (Fig. 2a-b). These results suggest that RSPO1 may induce the degradation of Axin1 via the ubiquitin–proteasome pathway.

### CK1ε is involved in Axin1 degradation mediated by RSPO1

**Fig. 3:**
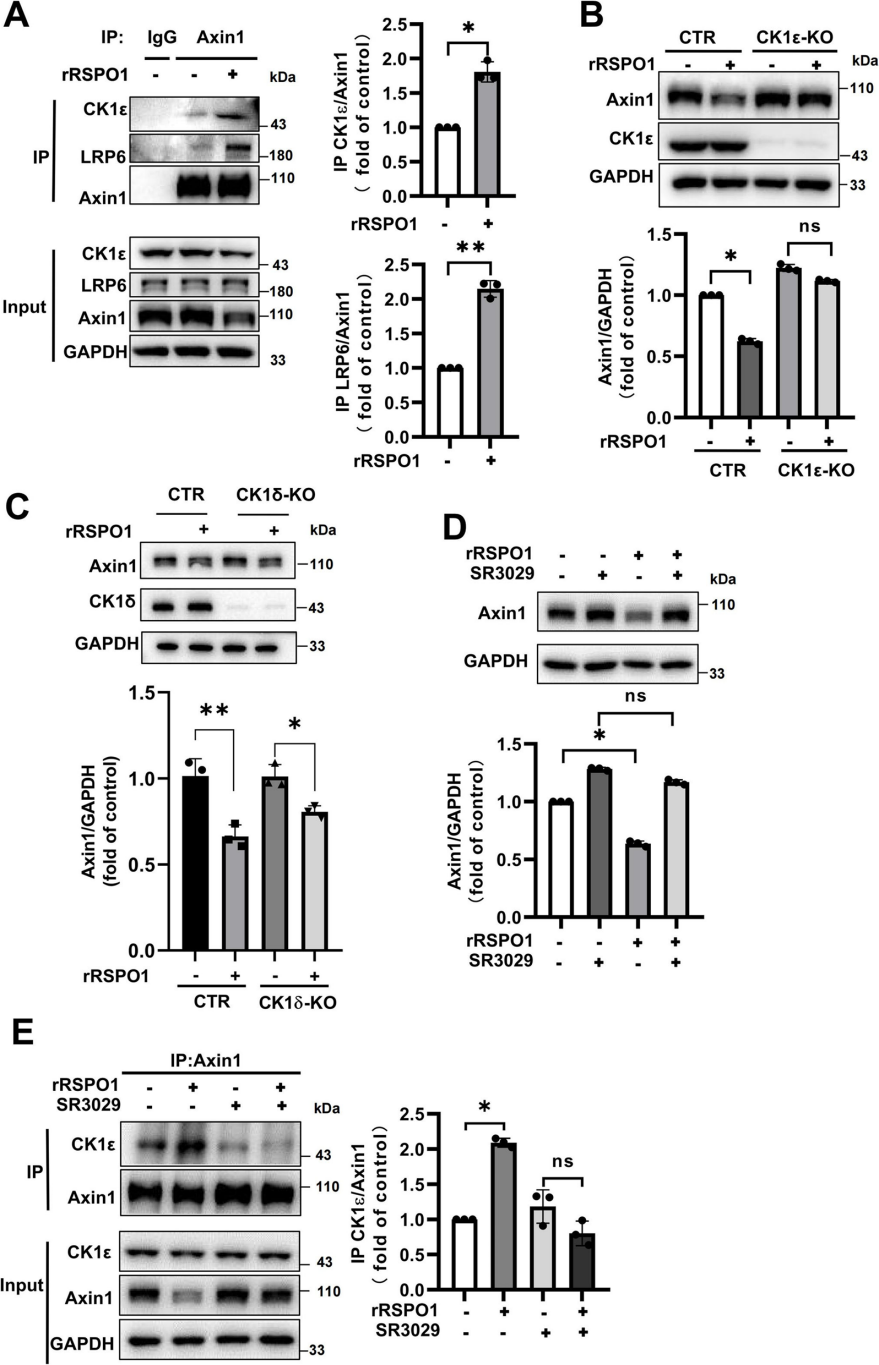
CK1ε is involved in Axin1 degradation mediated by RSPO1. **a** HEK293T cells were treated with 0.2 μg/mL rRSPO1 for 12 h. The interaction among endogenous Axin1, CK1ε and LRP6 was determined by immunoblotting. The data shown are representative of three independent experiments. The levels of CK1ε or LRP6 were quantitated by densitometry and normalized to Axin1. Quantitative results are displayed on the right. **b** CK1ε knockout (CK1ε-KO) cells or (**c**) CK1δ knockout (CK1δ-KO) cells and the control cells (CTR) or were treated with 0.2 μg/mL rRSPO1 for 12 h, and the protein expression levels of endogenous Axin1 and CK1ε/δ were determined by immunoblotting. The data shown are representative of three independent experiments. The levels of Axin1 were quantitated by densitometry and normalized to GAPDH. Quantitative results are displayed at the bottom. **d** HEK293T cells were treated with 0.2 μM SR3029 alone or in combination with 0.2 μg/mL rRSPO1 for 12 h, followed by Western blotting to determine the protein expression levels of endogenous Axin1. The data shown are representative of three independent experiments. The levels of Axin1 were quantitated by densitometry and normalized to GAPDH. Quantitative results are displayed at the bottom. **e** HEK293T cells were treated with 0.2 μg/mL rRSPO1 alone or with 0.2 μM SR3029 for 12 h, and the interaction of endogenous Axin1 with CK1ε was determined by immunoblotting. The data shown are representative of three independent experiments. The levels of CK1ε were quantitated by densitometry and normalized to Axin1. Quantitative results are displayed on the right. Data are presented as the mean ± SEM, *p < 0.05, **p < 0.01, *n* = 3

To explore the molecular mechanism of RSPO1-mediated Axin1 degradation, a coimmunoprecipitation (Co-IP) experiment was used to examine whether recombinant RSPO1 has any effect on the interaction of Axin1 with its associated proteins. The Co-IP results showed that treatment with RSPO1 significantly promoted the association of Axin1 with CK1ε and LRP6, suggesting that CK1ε may be involved in RSPO1-induced degradation of Axin1 (Fig. 3a). We next evaluated the role of CK1ε and CK1δ in the stability of the Axin1 protein. The CK1ε and CK1δ genes (CSNK1E and CSNK1D) were knocked out in HEK293T cells using CRISPR–Cas9 gene editing technology. As shown in Fig. 3b, knockout of CSNK1E resulted in an increase in Axin1 protein levels and remarkably attenuated the RSPO1-induced decrease in Axin1 protein levels, whereas CK1δ deficiency had little effect on the RSPO1-induced decrease in Axin1 protein levels (Fig. 3c), suggesting that CK1ε, but not CK1δ, may play a major role in RSPO-mediated downregulation of Axin1 expression in HEK293T cells.

Consistently, a specific CK1δ/CK1ε inhibitor, SR3029, also increased the protein level of Axin1 in HEK293T cells and blocked the decrease in Axin1 protein induced by RSPO1 (Fig. 3d). Moreover, SR3029 blocked RSPO1 promotion of the interaction between Axin1 and CK1ε, as demonstrated by a Co-IP assay (Fig. 3e). These results suggest that CK1ε may play an important role in RSPO-mediated stability of Axin1.

### RSPO1 induces Axin1 degradation via an LRP6/CK1ε-mediated mechanism

**Fig. 4:**
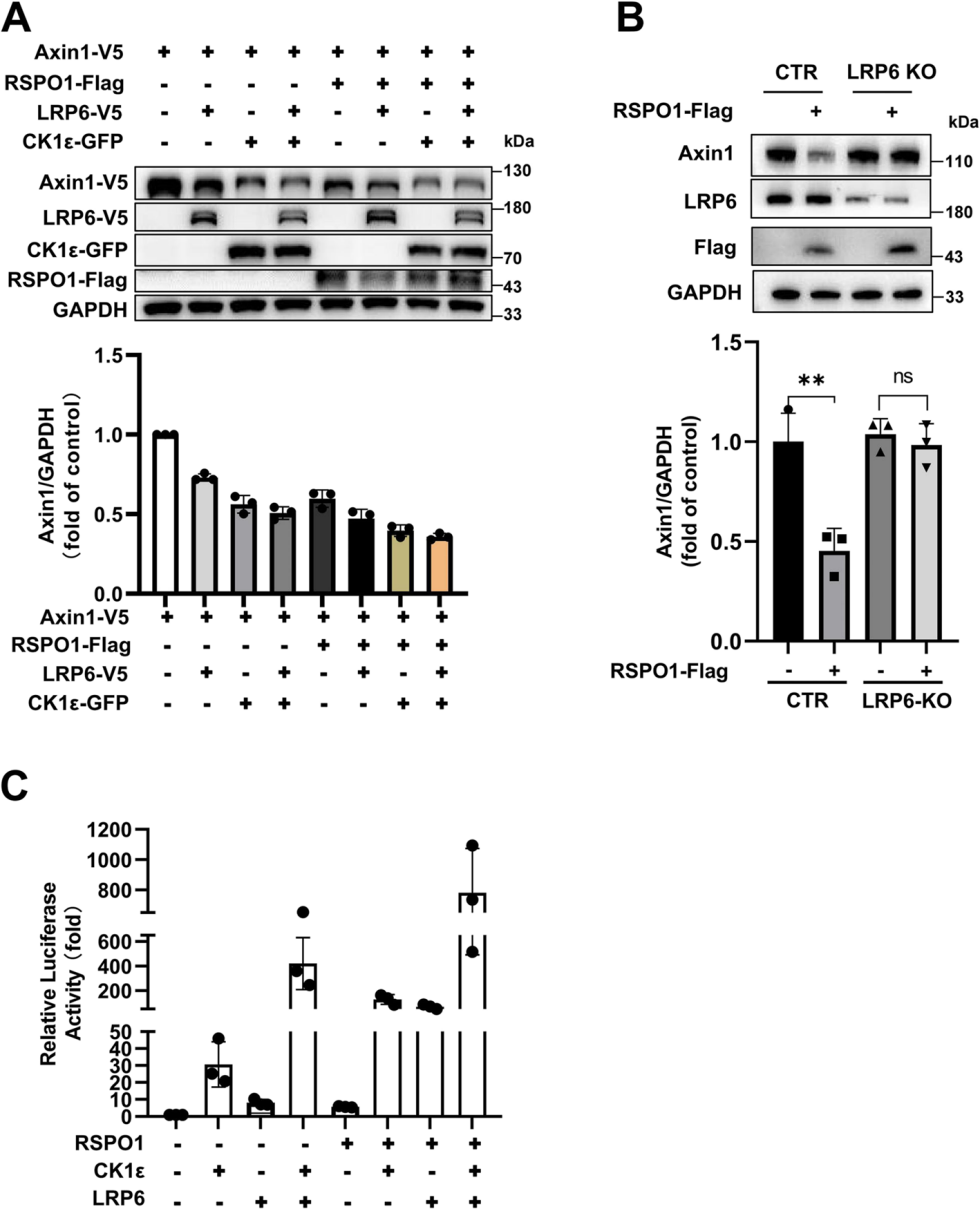
RSPO1, CK1ε and LRP6 have a synergistic effect on the degradation of Axin1 and Wnt/β-catenin signaling. **a** HEK293T cells were transfected with Axin1-V5 expression vector along with vector or expression plasmids for LRP6-V5, CK1ε-GFP and RSPO1-Flag. The expression of Axin1-V5, LRP6-V5, CK1ε-GFP and RSPO1-Flag was visualized by immunoblotting. The data shown are representative of three independent experiments. The levels of Axin1 were quantitated by densitometry and normalized to GAPDH. Quantitative results are displayed at the bottom. **b** LRP6-deficient (LRP6-KO) HEK293T cells and control cells (CTR) were transfected with a plasmid encoding Flag-tagged RSPO1, and the protein expression levels of Axin1, LRP6 and RSPO1-Flag were determined by immunoblotting. The data shown are representative of three independent experiments. The levels of Axin1 were quantitated by densitometry and normalized to GAPDH. Quantitative results are displayed at the bottom. **c** The SuperTOPFlash luciferase reporter gene was cotransfected into HEK293T cells with expression plasmids for RSPO1-Flag, CK1ε-GFP and LRP6-V5. The data were collected from three separate experiments. The luciferase values were normalized to β-gal activities and expressed as fold induction relative to the basal activity. Data are presented as the mean ± SEM, *p < 0.05, **p < 0.01, *n* = 3

The Wnt coreceptor LRP5/6 has been implicated in the degradation of Axin [29–32]. We next assessed the effect of LRP6 on RSPO1-induced degradation of Axin1. As demonstrated in Fig. 4a, similar to RSPO1, ectopic expression of LRP6 and CK1ε induced Axin1 degradation in HEK293 cells. The expression of LRP6 further potentiated Axin1 degradation mediated by RSPO1 or CK1ε (Fig. 4a). The LRP6 gene was knocked out in HEK293T cells using CRISPR–Cas9 gene editing technology. LRP6 deficiency remarkably attenuated the RSPO1-induced decrease in Axin1 protein, indicating the importance of LRP6 in RSPO1-induced downregulation of Axin1 expression (Fig. 4b). Moreover, the SuperTOPFlash reporter gene assay was used to examine the effect of RSPO1, CK1ε and LRP6 on Wnt/β-catenin signaling. Our results showed that simultaneous expression of RSPO1, CK1ε and LRP6 synergistically promoted the transcriptional activity of the SuperTOPFlash reporter (Fig. 4c). These results suggest that RSPO-induced accumulation of LRP6 was involved in Axin1 degradation in a CK1ε-dependent manner, and this mechanism might at least partially contribute to the activation of Wnt/β-catenin signaling.

### RSPO1 synergizes with Wnt3A to downregulate Axin1 expression

**Fig. 5:**
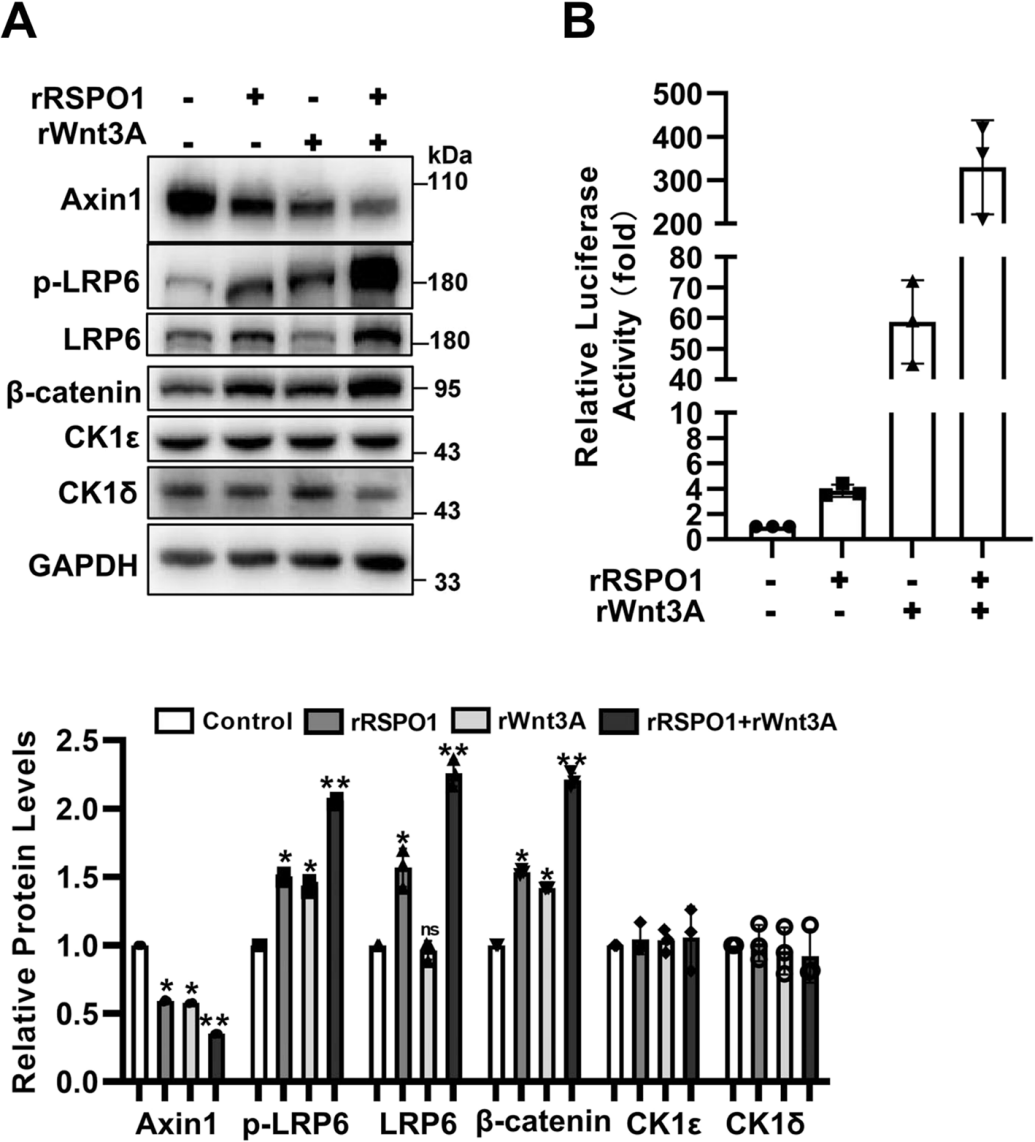
RSPO1 and Wnt3A have a synergistic effect on Axin1 degradation. **a** HEK293T cells were treated with 0.2 μg/mL rRSPO1 alone or in combination with 0.2 μg/mL recombinant Wnt3A protein (rWnt3A) for 12 h, and the protein expression of endogenous Axin1, LRP6, p-LRP6, β-catenin, CK1ε and CK1δ was determined by immunoblotting. The data shown are representative of three independent experiments. The protein levels from Western blotting were quantitated by densitometry and normalized to GAPDH. Quantitative results are displayed at the bottom. **b** HEK293T cells transfected with the SuperTOPFlash reporter gene were treated with 0.2 μg/mL rRSPO1 and/or 0.2 μg/mL rWnt3A for 24 h. The luciferase values were normalized to β-gal activities. Each treatment was performed in three replicates. Data are presented as the mean ± SEM, *p < 0.05, **p < 0.01, *n* = 3

RSPOs can synergize with Wnt3A to activate the Wnt/β-catenin signaling pathway [7, 8]. We tested the effect of RSPO1 and Wnt3A on the degradation of Axin1. HEK293T cells were treated with recombinant RSPO1 protein alone or in combination with purified Wnt3A protein. The immunoblotting results showed that RSPO1 and Wnt3A cooperatively downregulated the protein expression of Axin1, accompanied by the upregulation of LRP6, p-LRP6 and β-catenin expression (Fig. 5a). Moreover, combined treatment with recombinant RSPO1 and Wnt3A synergistically increased the activity of the SuperTOPFlash luciferase reporter (Fig. 5b). These results support the idea that RSPO1-mediated degradation of Axin1 may be a result of its function in promoting Wnt/β-catenin signaling.

### RSPOs mediate the stability of Axin1 and Axin2 via a similar mechanism

**Fig. 6:**
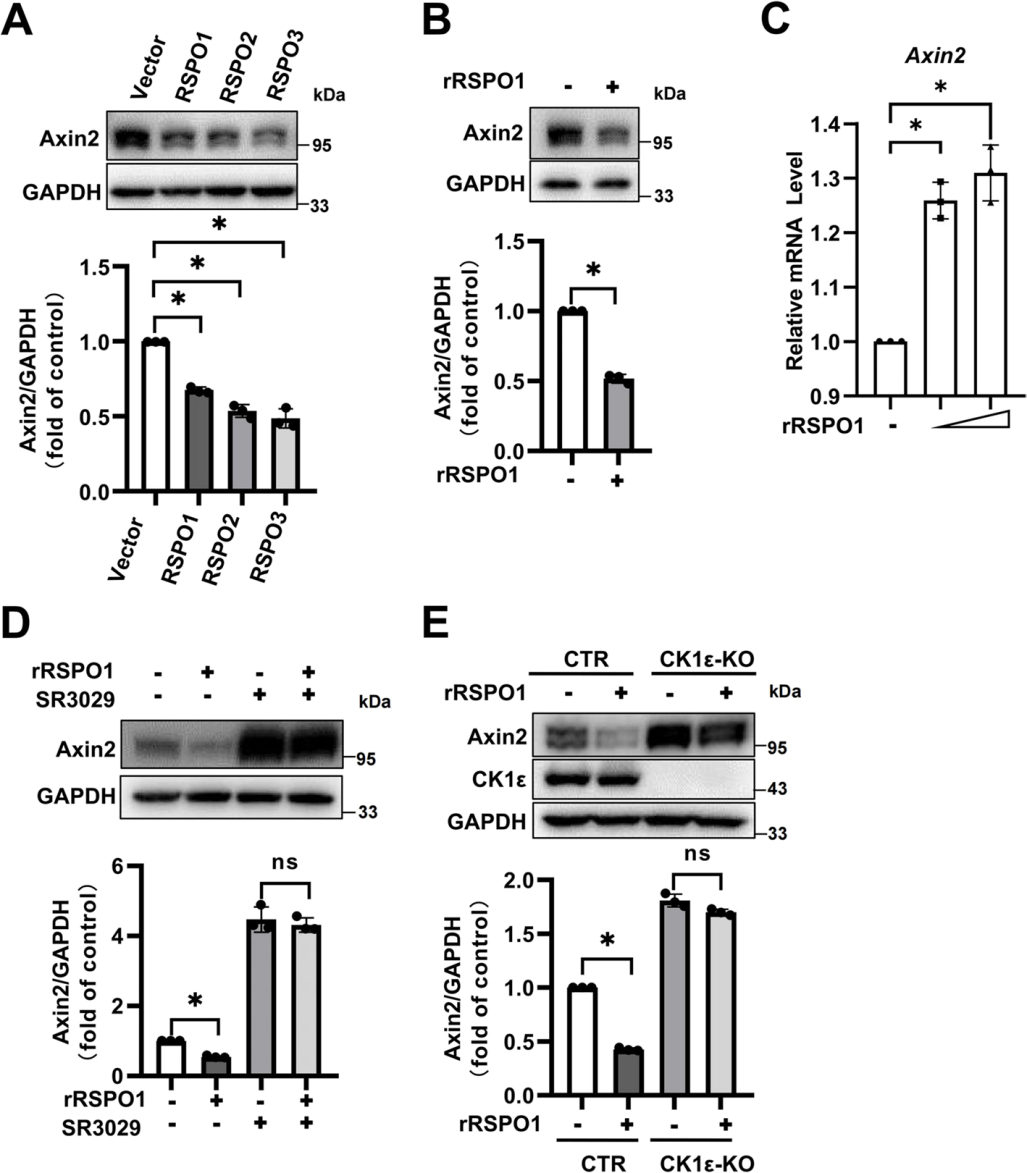
CK1ε is involved in Axin2 degradation mediated by RSPO1. **a** The plasmids encoding RSPO1-Flag, RSPO2-Flag and RSPO3-Flag were transfected into HEK293T cells, and the protein expression of endogenous Axin2 was determined by immunoblotting. The data shown are representative of three independent experiments. The levels of Axin2 were quantitated by densitometry and normalized to GAPDH. Quantitative results are displayed at the bottom. **b** HEK293T cells were treated with 0.2 μg/mL rRSPO1 for 12 h, and the protein expression of endogenous Axin2 was determined by immunoblotting. The data shown are representative of three independent experiments. The levels of Axin2 were quantitated by densitometry and normalized to GAPDH. Quantitative results are displayed at the bottom. **c** HEK293T cells were treated with a concentration gradient of rRSPO1 (0.2 and 0.4 μg/mL) for 12 h, followed by the measurement of mRNA levels of *Axin2* by real-time quantitative PCR. **d** HEK293T cells were treated with 0.2 μM SR3029 alone or in combination with 0.2 μg/mL rRSPO1 for 12 h, followed by Western blotting to measure the protein expression of endogenous Axin2. The data shown are representative of three independent experiments. The levels of Axin2 were quantitated by densitometry and normalized to GAPDH. Quantitative results are displayed at the bottom. (E) Control cells (CTR) or CK1ε knockout (CK1ε-KO) cells were treated with 0.2 μg/mL rRSPO1 for 12 h, and the levels of endogenous Axin2 and CK1ε were determined by immunoblotting. The data shown are representative of three independent experiments. The levels of Axin2 were quantitated by densitometry and normalized to GAPDH. Quantitative results are displayed at the bottom. Data are presented as the mean ± SEM, *p < 0.05, n = 3

Axin2 is a homolog of Axin1, and both Axin1 and Axin2 function to inhibit Wnt/β-catenin signaling [33]. Thus, we next examined the effect of RSPOs on the expression of Axin2 in HEK293T cells. The expression vectors encoding RSPO1, RSPO2 and RSPO3 were transfected into HEK293T cells, and the protein expression level of Axin2 was determined by Western blotting. The results showed that all three RSPOs dramatically reduced the protein level of Axin2 (Fig. 6a). Consistently, a decreased Axin2 protein level was observed following treatment with recombinant RSPO1 protein (Fig. 6b). However, as was demonstrated by real-time PCR, recombinant RSPO1 protein upregulated the mRNA expression of *Axin2* (Fig. 6c). These results indicate that RSPOs could regulate the stability of the Axin2 protein.

We then determined whether CK1ε is involved in RSPO-induced downregulation of Axin2 protein expression. As shown in Fig. 6d, the protein expression level of Axin2 was significantly increased in HEK293T cells with knockout of CK1ε, and CK1ε knockout at least partially blocked RSPO1-induced degradation of Axin2 (Fig. 6d). Similarly, SR3029 treatment also markedly increased the protein level of Axin2 and attenuated the effect of RSPO1 on the expression of Axin2 (Fig. 6e). Taken together, our results suggest that RSPOs may mediate the stability of Axin1 and Axin2 via a similar mechanism.

## Discussion

We have known for many years that Wnt3A can induce Axin1 degradation through a proteasome pathway [34]. The binding of Wnt3A to the receptor Frizzled and the coreceptor LRP5/6 promotes the interaction between LRP5/6 and Axin, which may initiate the degradation of Axin [29]. Wnt signaling through LRP5/6 promotes the degradation of Axin, leading to inhibition of the β-catenin destruction complex [29]. The Drosophila LRP5/6 ortholog Arrow has been shown to activate the Wnt signaling pathway by downregulating steady-state Axin expression [31]. In cultured mammalian cells, the expression of LRP5 promoted Axin degradation [29]. Kofron et al. reported that LRP6 was involved in Axin degradation in both oocytes and early embryos [32]. The LRP5 and LRP6 mutants lacking the extracellular domain destabilized Axin and promoted Axin degradation [29, 30]. On the other hand, stimulation of Wnt3A promoted the interaction between Axin and GSK3β by recruiting the E3 ubiquitin ligase SIAH to bind to Axin, resulting in ubiquitination and degradation of Axin [35, 36]. Although RSPO family members can activate the Wnt pathway by stabilizing the receptor Frizzled and the coreceptor LRP5/6 [6], whether RSPOs affect the stability of Axin has never been determined. In this study, we demonstrated that RSPO1 could induce the degradation of Axin1 via the ubiquitin–proteasome pathway. Stimulation with recombinant RSPO1 induced the accumulation of LRP6 and promoted the interaction among LRP6, CK1ε and Axin1, resulting in the degradation of Axin1. In this study, we revealed a novel mechanism by which RSPOs potentiate the Wnt signaling pathway through LRP6/CK1ε-mediated degradation of Axin1.

The recruitment of the Axin complex to the LRP5/6 receptor complex is a crucial event in Wnt signaling transduction. CK1ε is a well-known positive regulator of the Wnt signaling pathway, and the interaction between CK1ε and Axin1 has been reported in several previous studies [26, 37–39]. Axin1 has been shown to bind CK1ε in a protein phosphatase 2A (PP2A)- and Dishevelled-independent manner [26]. The carboxyl terminal domain of CK1ε is important for its binding to Axin [37]. Zhang et al. reported that the carboxyl terminal sequence and the MEKK1-interacting domain (MID) of Axin were needed for the binding of CK1ε to Axin and that the region of amino acids 248 to 295 in the CK1ε kinase domain had an important influence on the Axin-CK1ε interaction [38]. Harnos et al. identified three CK1ε-binding epitopes in the Axin1 sequence by peptide microarray screening [39]. These epitopes were located at amino acid residues 154–175, 274–295 and 592–616 [39]. The peptides corresponding to these epitopes specifically blocked the interaction between CK1ε and Axin1 [39]. In this study, we noted that treatment of HEK293T cells with recombinant RSPO1 significantly promoted the interaction between Axin1 and CK1ε. To further confirm whether CK1ε was involved in RSPO1-induced degradation of Axin1, we knocked out CK1ε in HEK293T cells. Knockout of the CK1ε gene caused an obvious increase in the Axin1 protein level and remarkably attenuated the RSPO1-induced decrease in Axin1 protein levels. Consistent with this result, the CK1δ/CK1ε inhibitor SR3029 also increased the protein level of Axin1 in HEK293T cells and blocked the RSPO1-induced interaction between Axin1 and CK1ε. We further showed that RSPO1 promoted the interaction among LRP6, CK1 and Axin1. Our results suggest that formation of the LRP6-CK1-Axin1 complex may trigger the degradation of Axin1. However, how this complex initiates Axin1 degradation remains to be determined.

There are two Axin isoforms, Axin1 and Axin2, in vertebrates. Axin1 was found to be constitutively expressed, while Axin2 expression was regulated by Wnt/β-catenin signaling [40–42]. Both proteins contain similar functional domains, including regulation of the G-protein signaling (RGS) domain that binds APC [43, 44], the disheveled and axin (DIX) domain [45, 46], and the binding sites for β-catenin and GSK3β [43, 44, 47–49]. Both Axin1 and Axin2 have interchangeable and redundant functions in the modulation of Wnt/β-catenin signaling. As expected, our results showed that recombinant RSPO1 could upregulate the mRNA expression of *Axin2* but had little effect on the transcription of Axin1 in HEK293T cells. Our results demonstrated that RSPOs could modulate the stability of Axin1 and Axin2 at the protein level. Either knockout of the CK1ε gene or SR3029 treatment markedly increased the protein level of Axin2 and attenuated the effect of the RSPO1 protein on the expression of Axin2, suggesting that RSPO1 may mediate the degradation of Axin1 and Axin2 through a similar mechanism.

In conclusion, in our study, we demonstrated that RSPOs could regulate Axin stability via the ubiquitin–proteasome pathway. Stimulation of RSPOs promoted the interaction among LRP6, CK1ε and Axin, thereby facilitating the formation of the LRP6-CK1ε-Axin complex, which may be important for the initiation of Axin degradation. Our results reveal that RSPOs mediate the degradation of Axin via a mechanism involving the LRP6-CK1ε axis.

### Supplementary Information

**Additional file 1: Figure S1**. rRSPO1 promotes the degradation of Axin1. HEK293T cells were treated with recombinant RSPO1, RSPO2, and RSPO3 proteins (rRSPO1, rRSPO2, and rRSPO3) at 0.2 μg/mL for 12 h. The protein expression levels of endogenous Axin1, LRP6, and β-catenin were measured by Western blotting.

## Data Availability

Data that support the findings of this study are available from the corresponding author upon reasonable request.

## Abbreviations

*RSPO*: R-spondin
*CK1*: Casein kinase 1
*GSK3β*: Glycogen synthase kinase 3β
*APC*: Adenomatous polyposis coli
*LRP6*: Low-density lipoprotein receptor-related protein 6
*DVL*: Disheveled
*AES*: Amino-terminal enhancer of split
*TMEM97*: Transmembrane protein 97
*LGR*: G-protein coupled receptor
*TSP*: Thrombospondin
*RNF43*: Ring finger protein 43
*ZNRF3*: Zinc and ring finger 3
*PP2A*: Phosphatase 2A
*DIX*: Disheveled and axin
*MID*: MEKK1-interacting domain
*RGS*: Regulator of G-protein signaling
