# Supplementary material for: R-spondin-1 induces Axin degradation via the LRP6-CK1ε axis
Source: Cell Commun Signal. 2024 Jan 5;22:14. doi: 10.1186/s12964-023-01456-y (PMC10768284; doi:10.1186/s12964-023-01456-y)
Supplement: Supplementary file 1 — Additional file 1: Figure S1. rRSPO1 promotes the degradation of Axin1. HEK293T cells were treated with recombinant RSPO1, RSPO2, and RSPO3 proteins (rRSPO1, rRSPO2, and rRSPO3) at 0.2 μg/mL for 12 h. The protein expression levels of endogenous Axin1, LRP6, and β-catenin were measured by Western blotting. [file 12964_2023_1456_MOESM1_ESM.docx]

**Supplementary Figure 1
Supplementary Figure 1.** **rRSPO1 promotes the degradation of Axin1.** HEK293T cells were treated with recombinant RSPO1, RSPO2, and RSPO3 proteins (rRSPO1, rRSPO2, and rRSPO3) at 0.2 μg/mL for 12 h. The protein expression levels of endogenous Axin1, LRP6, and β-catenin were measured by Western blotting.


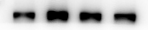

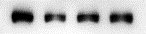

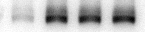


**rRSPO1**

**rRSPO2**

**rRSPO3**

**-**

**Axin1**

**LRP6**

**β-catenin**

**GAPDH**


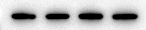


**kDa**

**110**

**180**

**95**

**33**
